# β-Catenin-Independent Activation of TCF1/LEF1 in Human Hematopoietic Tumor Cells through Interaction with ATF2 Transcription Factors

**DOI:** 10.1371/journal.pgen.1003603

**Published:** 2013-08-15

**Authors:** Luca Grumolato, Guizhong Liu, Tomomi Haremaki, Sathish Kumar Mungamuri, Phyllus Mong, Gal Akiri, Pablo Lopez-Bergami, Adriana Arita, Youssef Anouar, Marek Mlodzik, Ze'ev A. Ronai, Joshua Brody, Daniel C. Weinstein, Stuart A. Aaronson

**Affiliations:** 1 Department of Oncological Sciences, Icahn School of Medicine at Mount Sinai, New York, New York, United States of America; 2 INSERM U982, DC2N, Institute for Research and Innovation in Biomedicine, University of Rouen, Mont Saint Aignan, France; 3 Biology Department, Queens College of the City University of New York, Flushing, New York, United States of America; 4 Instituto de Medicina y Biología Experimental, CONICET, Buenos Aires, Argentina; 5 Department of Medicine, Hematology and Medical Oncology, Icahn School of Medicine at Mount Sinai, New York, New York, United States of America; 6 Department of Developmental and Regenerative Biology, Icahn School of Medicine at Mount Sinai, New York, New York, United States of America; 7 Signal Transduction Program, Sanford-Burnham Medical Research Institute, La Jolla, California, United States of America; Cincinnati Children's Hospital Medical Center, United States of America

## Abstract

The role of Wnt signaling in embryonic development and stem cell maintenance is well established and aberrations leading to the constitutive up-regulation of this pathway are frequent in several types of human cancers. Upon ligand-mediated activation, Wnt receptors promote the stabilization of β-catenin, which translocates to the nucleus and binds to the T-cell factor/lymphoid enhancer factor (TCF/LEF) family of transcription factors to regulate the expression of Wnt target genes. When not bound to β-catenin, the TCF/LEF proteins are believed to act as transcriptional repressors. Using a specific lentiviral reporter, we identified hematopoietic tumor cells displaying constitutive TCF/LEF transcriptional activation in the absence of β-catenin stabilization. Suppression of TCF/LEF activity in these cells mediated by an inducible dominant-negative TCF4 (DN-TCF4) inhibited both cell growth and the expression of Wnt target genes. Further, expression of TCF1 and LEF1, but not TCF4, stimulated TCF/LEF reporter activity in certain human cell lines independently of β-catenin. By a complementary approach *in vivo*, TCF1 mutants, which lacked the ability to bind to β-catenin, induced *Xenopus* embryo axis duplication, a hallmark of Wnt activation, and the expression of the Wnt target gene *Xnr3*. Through generation of different TCF1-TCF4 fusion proteins, we identified three distinct TCF1 domains that participate in the β-catenin-independent activity of this transcription factor. TCF1 and LEF1 physically interacted and functionally synergized with members of the activating transcription factor 2 (ATF2) family of transcription factors. Moreover, knockdown of ATF2 expression in lymphoma cells phenocopied the inhibitory effects of DN-TCF4 on the expression of target genes associated with the Wnt pathway and on cell growth. Together, our findings indicate that, through interaction with ATF2 factors, TCF1/LEF1 promote the growth of hematopoietic malignancies in the absence of β-catenin stabilization, thus establishing a new mechanism for TCF1/LEF1 transcriptional activity distinct from that associated with canonical Wnt signaling.

## Author Summary

The Wnt signaling pathway plays a crucial role during embryonic development and in the maintenance of stem cell populations in various organs and tissues. Aberrant activation of this pathway through different mechanisms participates in the onset and progression of several types of human cancers. In the presence of Wnt ligands, stabilized β-catenin acts as a transcriptional activator to induce the expression of target genes through binding to the TCF/LEF family of transcription factors. Using *in vitro* and *in vivo* models, we show that TCF/LEF proteins can be activated independently of β-catenin through cooperation with members of the ATF2 subfamily of transcription factors. This novel alternative mechanism of TCF/LEF activation is constitutively up-regulated in certain hematopoietic tumor cells, where it regulates the expression of TCF/LEF target genes and promotes cell growth.

## Introduction

The Wnt/β-catenin signaling pathway plays an essential role during embryonic development and as a major regulator of stem/progenitor cell maintenance in a number of postnatal organs and tissues, including the gastrointestinal tract, the skin and the hematopoietic system [1]–[3]. Genetic alterations that lead to aberrant activation of this pathway occur very commonly in certain tumors, including colon cancer, hepatocellular carcinomas and adrenocortical adenoma [4], [5]. In other types of tumors, alternative mechanisms are more frequently responsible for Wnt/β-catenin constitutive up-regulation. Indeed, a Wnt autocrine transforming activity was initially discovered in the mouse model three decades ago [6], and we have established that this mechanism also occurs frequently in different human cancers, including breast cancer [7], non small cell lung cancer [8] and sarcoma [9].

The Wnt/β-catenin, or canonical, pathway is initiated by Wnt-mediated coupling of the seven transmembrane domain receptor Frizzled and the single-membrane-spanning low-density receptor-related protein 5/6 (LRP5/6), followed by phosphorylation of the LRP5/6 intracellular domain [10], [11]. Through a mechanism not yet fully understood, phosphorylated LRP5/6 leads to the inhibition of the so-called β-catenin destruction complex, composed of axin, glycogen synthase kinase 3, dishevelled (Dvl), casein kinase 1 and the tumor suppressor adenomatous polyposis coli, resulting in the accumulation of β-catenin in the cytoplasm and the nucleus [11], [12]. In the absence of Wnt activation, the four members of the TCF/LEF family of transcription factors form a complex with Groucho/TLE repressors and inhibit gene expression [3], [13], [14], [15], [16], [17]. Through either direct competition [18] or XIAP-mediated ubiquitylation [19], nuclear β-catenin displaces Groucho/TLE and binds to TCF/LEF factors, thus promoting a transcriptional switch that allows the expression of Wnt target genes, including *Myc*, *cyclin D1*, *Axin 2* and *Lef1* [4], [20].

While its role in the control of gene expression is thought to depend mainly on the interaction with TCF/LEF proteins, β-catenin can act in some contexts through binding to other transcription factors, including the homeodomain protein Prop1 [21], various nuclear receptors [17], [22], the forkhead box O factors [23] and the Krueppel-like factor 4 [24]. TCF/LEF factors can also interact with other proteins, including ALY [25], Smad [26], [27] and c-Jun [28], allowing the formation of large nuclear complexes that control the expression of genes containing a particular combination of regulatory sequences in their promoter/enhancer. While providing an additional level of regulation, such interactions do not challenge the general notion that nuclear β-catenin is required for TCF/LEF transcriptional activity, which is widely supported by biochemical and genetic data [2], [15], [29]. An exception to this paradigm may involve the hematopoietic system, where intriguing discrepancies in the phenotypes of TCF1/LEF1 and β-catenin null mice have suggested that in particular contexts canonical Wnt signals could be transduced independently of β-catenin [30]–[36].

While the β-catenin pathway has been reported to be upregulated in cancer stem cells of different leukemias [37]–[40] and in the transition from chronic to acute myelogenous leukemia [41], mutations in intracellular components that are frequently responsible for Wnt pathway activation in solid tumors are uncommon in hematopoietic tumors. Here we show that some hematopoietic tumor cells display β-catenin-independent TCF/LEF activity, whose down-regulation inhibits the expression of TCF/LEF target genes and cell growth. Using both *in vitro* and *in vivo* approaches, we further demonstrate that TCF1 retains transcriptional activity in the absence of β-catenin. Finally, we establish that ATF2 family members physically and functionally interact with TCF1/LEF1 factors to promote target gene expression and hematopoietic tumor cell growth. Together, our results uncover a new mechanism that activates TCF1/LEF1 transcriptional activity independently of β-catenin and the Wnt canonical pathway.

## Results

### β-catenin-independent TCF/LEF activity in hematopoietic tumor cells

**Figure 1:**
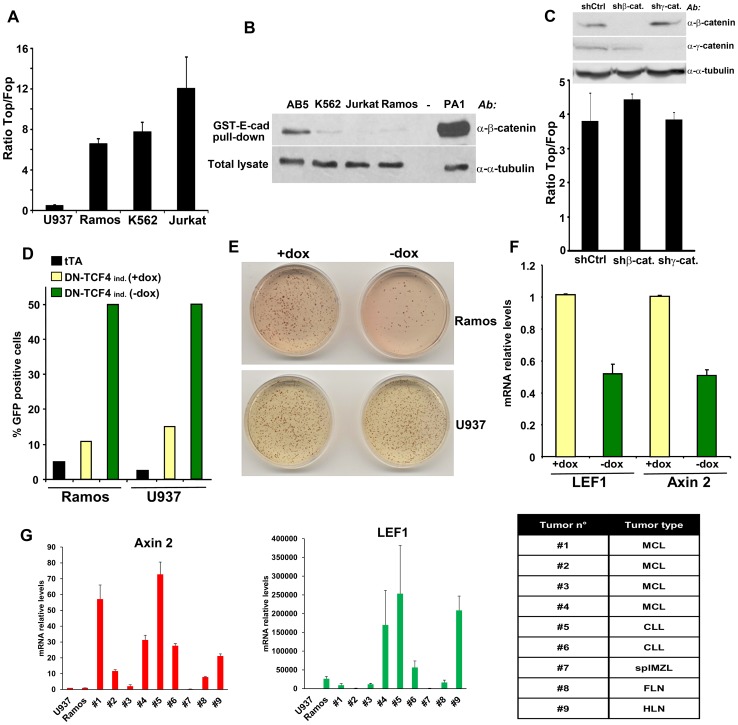
Constitutive TCF/LEF activity in hematopoietic tumor cells in the absence of β-catenin stabilization. (A) The indicated hematopoietic tumor cells were transduced with wild-type (Top) or mutant (Fop) TCF/LEF firefly luciferase reporter and a renilla luciferase virus, and the TCF/LEF transcriptional activity was calculated by dividing the TOP/renilla ratio by the FOP/renilla ratio. The mean values (± s.e.m., n>5) of at least two independent experiments are shown. (B) Lack of β-catenin stabilization in K562, Jurkat and Ramos cells, as measured by GST-E-cadherin pull-down. Ovarian PA1 cancer cells and breast immortalized AB5 cells were used as positive and negative control, respectively. (C) β-catenin or γ-catenin knockdown in Ramos cells did not affect TCF/LEF activity. Ramos cells containing Top or Fop luciferase reporter were infected with β-catenin or γ-catenin shRNA and the TCF/LEF reporter activity was measured (lower panel). β-catenin and γ-catenin down-regulation was assessed by immunoblot (upper panel). (D) Ramos and U937 cells containing the tet transactivator (tTA) with or without a lentiviral inducible vector containing DN-TCF4 fused to GFP were cultured for 24 hrs in the presence or the absence of doxycycline (dox), and the proportion of GFP positive cells was assessed by FACS analysis. (E) The cells in D were used in colony formation assay in the presence or the absence of dox. (F) Effects of DN-TCF4 induction in Ramos cells on the expression of LEF1 and Axin 2 by quantitative real-time PCR. (G) mRNA levels of Axin 2 and LEF1 in human primary hematopoietic tumors. For each sample, the qPCR values were normalized with those obtained for the negative control, U937 cells. The types of malignancy corresponding to each sample are listed in the table: MCL, mantle cell lymphoma; CLL, B-cell chronic lymphocytic leukemia; splMZL, splenic marginal zone lymphoma; FLN, follicular lymphoma; HLN, Hodgkin's lymphoma.

Using lentiviral wild-type (Top) and mutant (Fop) TCF/LEF reporters, we investigated the status of Wnt signaling in cells derived from several types of human cancer. As a result of this screen, we identified up-regulated TCF/LEF activity in different hematopoietic tumor cells, including Ramos, K562 and Jurkat (Figure 1A). As shown in Figure S1A, TCF/LEF reporter levels in these cells were comparable to those observed in solid tumor cells exhibiting autocrine Wnt activation [8], [9]. To assess the levels of stabilized, transcriptionally active β-catenin in these same hematopoietic tumor lines, we used an approach based on the capture of uncomplexed β-catenin with GST-E-cadherin beads [7]–[9]. Surprisingly, none showed detectable levels of stabilized β-catenin (Figure 1B). It has been reported that γ-catenin can also play a role in Wnt signaling, probably through a mechanism involving stabilization of β-catenin [42], [43]. However, neither down-regulation of β-catenin nor γ-catenin had any effect on the TCF/LEF reporter in Ramos and K562 cells (Figures 1C and S1B), indicating that β-catenin and γ-catenin were dispensable for TCF/LEF transcriptional activation in these hematopoietic tumor cells.

To gain insights into the biological relevance of this β-catenin-independent TCF/LEF activity, we tested the effects of a GFP-fused DN-TCF4 in Ramos lymphoma cells using a lentiviral tetracycline (Tet-off) inducible system. As a negative control for these experiments, we utilized U937 lymphoma cells, which lacked detectable constitutive TCF/LEF activity (Figure 1A). Doxycyline withdrawal triggered expression of DN-TCF4 to a similar extent in Ramos and U937 cells, as measured by FACS analysis (Figure 1D). Under these conditions, DN-TCF4 expression inhibited colony formation by Ramos cells but had no effect on U937 cells (Figure 1E). Of note, induction of DN-TCF4 expression in Ramos cells also reduced the mRNA levels of LEF1 and Axin 2 (Figure 1F), two well-established Wnt target genes. Consistent with these results, DN-TCF4 also inhibited TCF/LEF reporter activity as well as the expression of LEF1 and Axin 2 in K562 (Figures S1C and S1D). Together, these results indicated that some hematopoietic tumor lines exhibit elevated β-catenin-independent TCF/LEF activity, which positively influences the expression of Wnt target genes and tumor cell growth.

We extended our analysis to other hematopoietic tumor cell lines as well as primary tumors. Several mantle cell lymphoma lines, including Granta-519, HBL-2, JEKO-1 and JVM-2, exhibited TCF/LEF reporter levels as high or higher than Ramos (Figure S1E) and also contained undetectable levels of uncomplexed β-catenin (Figure S1F), implying their activation of β-catenin-independent TCF/LEF transcriptional activity. We also surveyed a series of primary hematopoietic tumors for expression of Axin 2 and LEF1 as compared to levels present in Ramos and U937 cells. Several exhibited high expression levels of these TCF/LEF target genes in the absence of detectable uncomplexed β-catenin (Figures 1G and S1G), arguing that activation of this novel pathway was not limited to hematopoietic tumor lines but occurred in primary human hematopoietic tumors as well.

### TCF1 and LEF1, but not TCF4, are transcriptionally active independently of β-catenin

**Figure 2:**
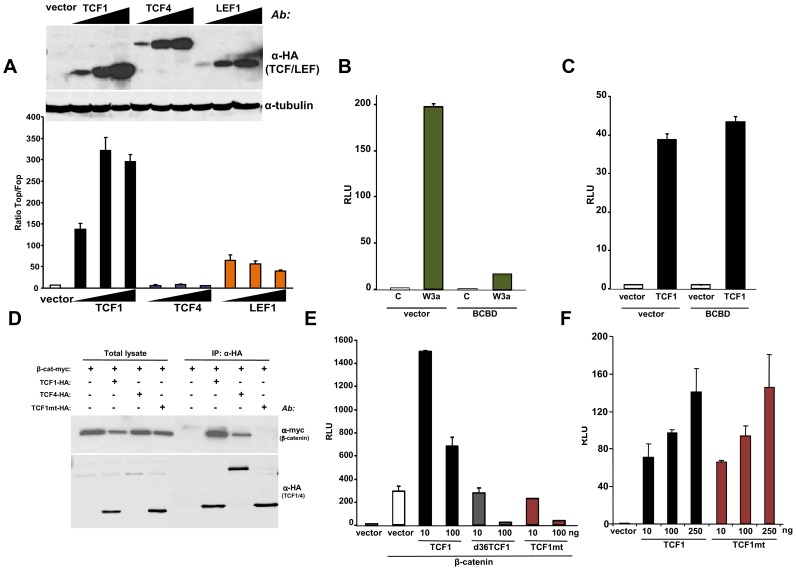
β-catenin-independent transcriptional activity of TCF1/LEF1 factors. (A) Empty pcDNA3HA vector or increasing amounts of HA-tagged TCF1, LEF1 or TCF4 constructs were co-transfected with SuperTop or SuperFop and renilla luciferase plasmids in 293T cells and the Top/Fop ratio was calculated. (B–C) Effects of a decoy construct containing TCF1 β-catenin binding domain (BCBD) on Wnt3a- and TCF1-induced TCF/LEF reporter activity. 293T cells were co-transfected with SuperTop and renilla reporter plasmids, together with the Wnt3a, TCF1 and BCBD vectors as indicated. (D–E) Mutant TCF1 (mtTCF1; D21A, E29K) is unable to bind to β-catenin. (D) 293T cells were transfected with the indicated HA-tagged TCF1 or TCF4 constructs and the myc-tagged β-catenin, followed by immunoprecipitation using anti-HA antibody and immunoblot with anti-myc or anti-HA antibodies. (E) 3T3 cells were co-transfected with β-catenin, the indicated amounts of TCF1, d36TCF1 or mtTCF1, together with SuperTop and renilla luciferase plasmids. (F) Effects of TCF1 and mtTCF1 expression on TCF/LEF reporter activity. 293T cells were co-transfected with SuperTop and renilla luciferase plasmids and different amounts of TCF1 or mtTCF1, followed by reporter assay.

TCF1 and LEF1 were cloned in 1991 from hematopoietic cells [44]–[46] and belong to a four-member family of transcription factors containing a N-terminal β-catenin binding domain and a high mobility group DNA binding domain located closer to the C-terminus [15]. To investigate whether these factors could act as transcriptional activators in the absence of β-catenin, we expressed TCF1, LEF1 or TCF4 in 293T cells, which lack constitutive Wnt activation. Figure 2A shows that TCF1 and LEF1, but not TCF4, stimulated TCF/LEF reporter activity in these cells. The stimulatory effect of TCF1 was observed using three different reporter constructs, containing respectively 2, 4 and 8 TCF/LEF repeats generated using different backbone vectors (Figure S2A). As an additional specificity control, TCF1-induced reporter activity was strongly inhibited by an excess of DN-TCF4 (Figure S2B). Of note, deletion of the first 65 aa, containing the β-catenin binding domain, only partially reduced the TCF/LEF activation induced by TCF1 (Figure 2A), indicating that β-catenin was not strictly required for this effect. To inhibit any residual nuclear β-catenin potentially present in 293T cells, we generated a decoy construct containing the TCF1 β-catenin binding domain fused to the Gal4 DNA binding domain (BCBD). While almost completely abolishing the up-regulation of the TCF/LEF reporter induced by Wnt3a (Figure 2B), BCBD did not affect TCF1 transcriptional activity (Figure 2C), strongly arguing against the involvement of β-catenin in this activity.

Based on the crystal structure of the β-catenin-TCF3 complex, we mutated two residues in the TCF1 β-catenin binding domain, D21A and E29K, which should impair the ability of this transcription factor to bind to β-catenin [47]. Figures 2D and S3A show that this mutant, designated TCF1mt, was unable to physically interact with β-catenin by co-immunoprecipitation and failed to synergize with β-catenin or γ-catenin to stimulate the Wnt responsive reporter, instead exerting an antagonistic effect (Figures 2E, S3B and S3C). In contrast, both wild type TCF1 and TCF1mt expression stimulated TCF/LEF activity in 293T cells to similar extents (Figure 2F), implying that TCF1 was able to act as a transcriptional activator independently of β-catenin.

### TCF1, but not TCF4, stimulates the expression of Wnt target genes in *Xenopus laevis* embryos independently of β-catenin

**Figure 3:**
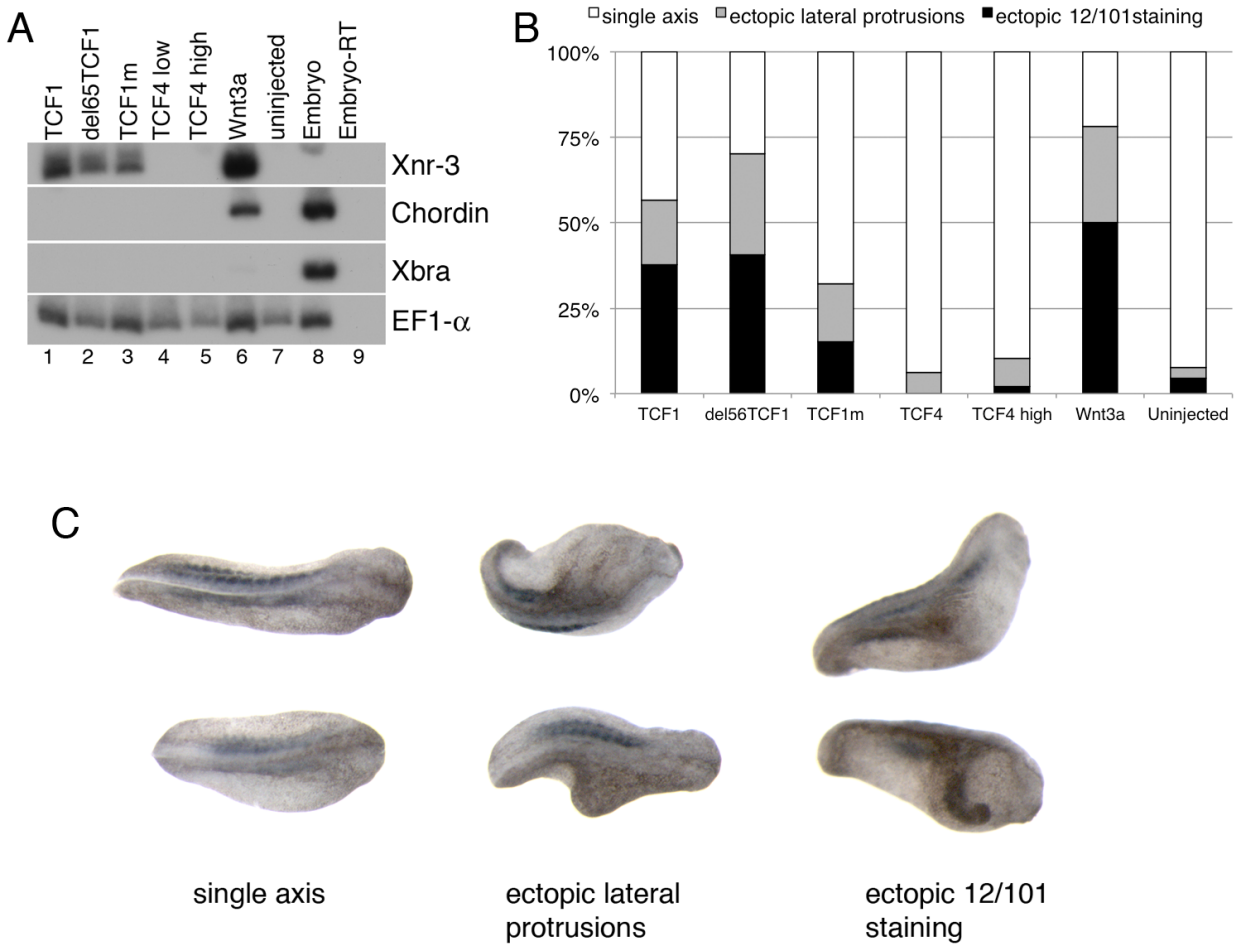
TCF1 activity in *Xenopus laevis* explants and embryos. (A) Wnt3a, wild-type and β-catenin-independent mutants of TCF1, but not TCF4, induce expression of the canonical Wnt pathway-responsive gene *Xnr3* in ectodermal (animal cap) explants. RT-PCR analysis of animal caps dissected at late blastula stages and cultured until stage 10.5. EF1-α is used as a loading control. The –RT lane contains all reagents except reverse transcriptase, and is used as a negative control. (B) Graph depicting embryonic perturbation by Wnt3a, TCF4, and wild-type and mutant TCF1. Embryos were injected in the ventral marginal zone at early cleavage stages and cultured until late tailbud stages. For (A) and (B), 1 ng (TCF4 high) or 500 pg (TCF1, del65TCF1, TCF1m, TCF4low, Wnt3a) of each RNA was injected, as listed. (C) Representative embryos recorded in the graph shown in (B). Dorsal views; anterior is to right. All embryos in (C) were injected with RNA encoding del65TCF1. Staining with the 12/101 antibody, which recognizes a somite specific epitope, was used to assess axis duplication.

During the earliest stages of *Xenopus* embryonic development, locally activated Wnt signaling induces the asymmetric expression of genes of the dorsal organizer, thus allowing the establishment of dorsal-ventral polarity [48]–[50]. We used this *in vivo* model to assess the β-catenin-independent activity of TCF1. As previously reported [51], injection of *tcf1*, but not *tcf4*, mRNA induced ectopic expression of the Wnt direct target gene *Xnr3* in animal cap explants (Figure 3A). Of note, we found that both TCF1mt and the N-terminal truncated TCF1 (del65TCF1) were able to trigger *Xnr3* expression, indicating that β-catenin was not required for this effect. Under these conditions, Wnt3a, but not the TCF1 constructs, increased the expression of another organizer gene, *Chordin*, likely reflecting its ability to induce a higher level of Wnt pathway activation. As a specificity control, neither TCF1 nor Wnt3a had an effect on the TGF-β target gene *Xbra* (Figure 3A). Consistent with these data, all TCF1 constructs, but not TCF4, induced axis duplication in the embryos (Figure 3B). Together, our findings indicate that in the absence of β-catenin TCF1 does not strictly act as a transcriptional repressor, but instead can stimulate the expression of Wnt canonical target genes and functions *in vivo* independently of β-catenin.

### Different TCF1 domains are involved in its β-catenin-independent transcriptional activity

To gain insights into the mechanisms responsible for β-catenin-independent TCF1/LEF1 transcriptional activity, we next investigated the contribution of different TCF1 domains to its ability to stimulate TCF/LEF reporter activity. Alternative splicing gives rise to several isoforms of each TCF/LEF protein, which in some cases can affect their activity [14], [15]. While the majority of these splicing events involve the C-terminus, experiments in *Xenopus* have suggested that the central exon IVa might be responsible for the balance between the activating and repressive functions of these transcription factors [52], [53]. We found that the presence or the absence of exon IVa did not affect TCF/LEF1 reporter activity induced by human TCF1 in 293T cells (Figure S4), indicating that this region is not involved in β-catenin-independent TCF1 transcriptional activity.

**Figure 4:**
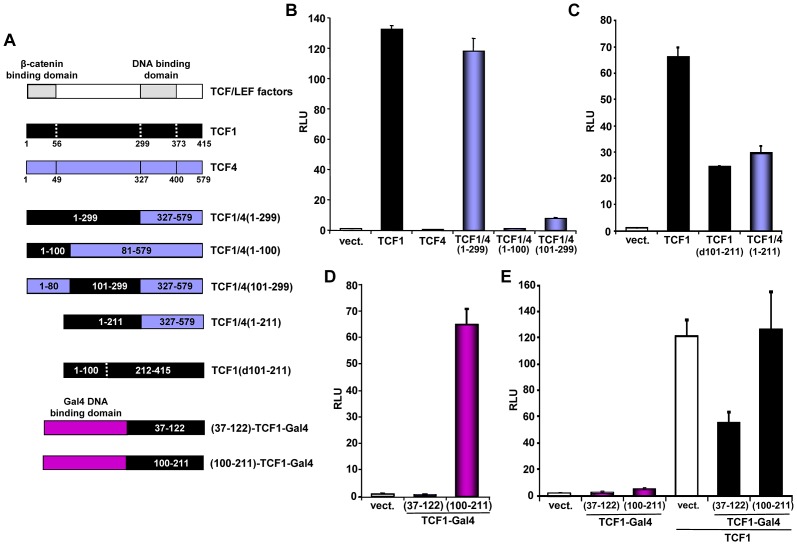
Mapping of the TCF1 domains involved in β-catenin-independent transcriptional activity. (A) Diagram of the different constructs used for the mapping. (B–D) 293T cells were co-transfected with the SuperTop reporter, the renilla plasmid and the indicated constructs, followed by reporter assay two days after transfection. (B) TCF1 N-terminal domain is required, but not sufficient, for β-catenin-independent full transcriptional activity. (C) Deletion of either aa 101–211 or 212–299 inhibits the β-catenin-independent transcriptional activity of TCF1. (D) Transcriptional activity of two different TCF1 domains fused to the Gal4 DNA binding domain. 293T cells were co-transfected with a Gal4 responsive reporter and the indicated TCF1-Gal4 fusion constructs, followed by reporter assay. (E) Expression of TCF1 aa 37–122, but not aa 100–211, inhibits TCF1-induced reporter activity. 293T cells were co-transfected with the Supertop reporter and the indicated TCF1-Gal4 fusion constructs, in the presence or the absence of TCF1, followed by reporter assay two days after transfection.

It has been reported that certain C-terminal TCF splice variants can bind to repressors, including CtBP [54], although the biological relevance of such interactions is controversial [15], [55]. To assess whether the β-catenin independent activity of TCF1 was associated with a particular C-terminal domain, we generated a chimeric protein containing the first 299 aa of TCF1 fused to the DNA-binding domain and C-terminus of TCF4 (TCF1/4(1–299); Figure 4A). We observed that TCF1 and TCF1/4(1–299) stimulated the TCF/LEF reporter to a similar extent, while TCF4 was inactive (Figure 4B), indicating that TCF1 C-terminus and DNA binding domains were not required for this effect. Using a similar approach, we showed that swapping the N-terminal 100 aa of TCF1 with the corresponding domain of TCF4 resulted in nearly inactive fusion proteins (Figure 4B). These results implied that this region was important for TCF1 transcriptional activity but not sufficient to confer β-catenin-independent transcriptional activity to TCF4. We extended TCF1 mapping and found that deletion of the region between aa 56/101 and aa 211 partially reduced TCF1 transcriptional activity in the absence of β-catenin (Figure 4C and S5A) but had no effect on the synergy between TCF1 and β-catenin (Figure S5B). As shown in Figure 4C, a TCF1/4 fusion protein containing TCF1 aa 1–211 and the TCF4 DNA binding domain and C-terminus showed decreased TCF/LEF activity, implying that aa 212–299 are also involved in β-catenin-independent TCF1 activity.

As a complementary approach, we assessed the activity of different TCF1 domains fused to the Gal4 DNA binding domain. Consistent with the results shown in Figures 4B and 4C, TCF1 aa 100–211 strongly up-regulated the activity of a Gal4 reporter, while aa 37–122 had no effect (Figure 4D). Of note, expression of TCF1(37–122)-Gal4, but not TCF1(100–211)-Gal4, inhibited TCF/LEF reporter activity induced by TCF1 in 293T cells (Figure 4E), suggesting that TCF1(37–122)-Gal4 may interfere with the binding to a TCF1 molecular partner, while TCF1(100–211) may have an intrinsic transactivation activity. Together, these results indicated that these three TCF1 domains have distinct roles in its β-catenin independent activity: the region between aa 100–211 is partially active on its own, while the N-terminal 100 aa and aa 211–299 are required for full transcriptional function.

### ATF2 transcription factors cooperate with TCF1/LEF1 to promote the growth of hematopoietic tumor cells

**Figure 5:**
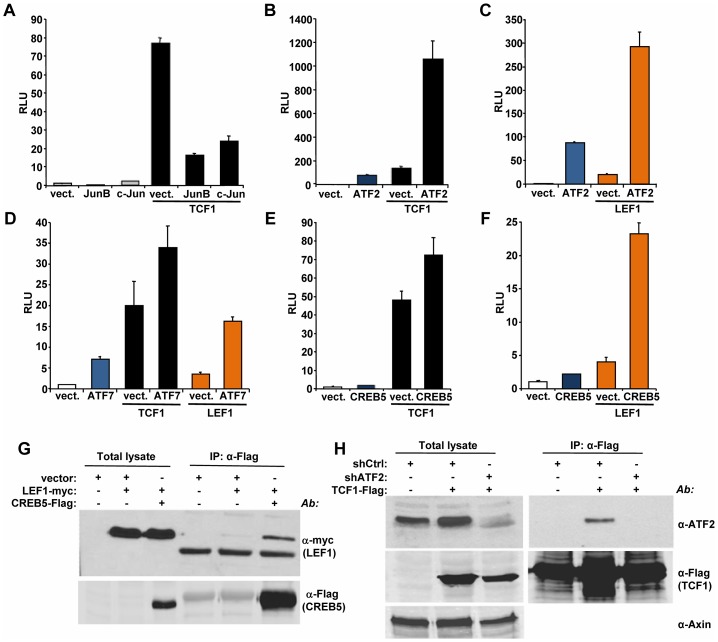
ATF2 transcription factors cooperate with TCF1/LEF1 to stimulate TCF/LEF activity. (A–F) 293T cells were co-transfected with the Supertop reporter, the renilla plasmid and the indicated TCF/LEF or AP1 transcription factors, followed by TCF/LEF reporter assay two days after transfection. (A) c-Jun and JunB inhibition of TCF1-induced reporter activity. (B–C) ATF2 synergizes with TCF1 (B) and LEF1 (C) to increase TCF/LEF activity. (D) ATF7 enhances TCF1- and LEF1-induced reporter activity. (E–F) CREB5 cooperated with TCF1 (E) and LEF1 (F). (G) myc-tagged LEF1 co-immunoprecipitates with Flag-tagged CREB5 in 293T cells. (H) Endogenous ATF2 co-immunoprecipitates with Flag-tagged TCF1 in 293T cells. Cells transduced with lentiviral shATF2 were used as a negative control and immunoblot with anti-Axin antibody was used as loading control for the total lysate.

We used a candidate approach to identify partners for TCF1/LEF1 potentially involved in their β-catenin independent activity. It has been shown that c-Jun binds to TCF4 and β-catenin to promote intestinal cancer development [28]. We tested the effects of TCF1 and c-Jun co-expression in 293T cells and found that c-Jun actually decreased TCF1-induced reporter activity (Figure 5A). A similar inhibitory effect was observed upon expression of the c-Jun-related JunB (Figure 5A), indicating that the c-Jun family has opposite effects in β-catenin-dependent and β-catenin-independent TCF/LEF signaling. These results prompted us to investigate the effects of other activator protein-1 (AP-1) factors and Jun binding partners on TCF1 transcriptional activity. While c-Fos had no effect (data not shown), ATF2 strongly synergized with TCF1 in stimulating the TCF/LEF reporter (Figure 5B). Of note, expression of ATF2 alone moderately increased reporter activity (Figure 5B), presumably through endogenous TCF1/LEF1. ATF2 showed a similar synergistic effect when co-expressed with TCF1mt, as well as in the presence of either Dvl or β-catenin shRNA (Figure S6), implying that the cooperation between TCF1 and ATF2 was independent of β-catenin. The fact that ATF2 also enhanced LEF1-induced reporter activity (Figure 5C) suggested that the synergy we uncovered between TCF1 and ATF2 could be part of a more general cooperation between the TCF1/LEF1 and ATF2 families of transcription factors. Indeed, expression of the two other ATF2-like proteins, i.e. activating transcription factor 7 (ATF7) and cAMP responsive element binding protein 5 (CREB5), also increased the TCF/LEF transcriptional activity of TCF1 and LEF1 (Figures 5D, 5E and 5F). Consistent with our previous findings, TCF4 was unable to stimulate the TCF/LEF reporter even in the presence of overexpressed ATF2, CREB5 or ATF7 (Figure S7A). Of note, various combinations of TCF1/LEF1 and ATF2 factors displayed different degrees of cooperation, with TCF1-ATF2 and LEF1-CREB5 showing the strongest synergy, likely reflecting preferential interactions among distinct members of these two transcription factor families.

To gain insights into the mechanisms involved in the synergistic functional interactions of TCF1/LEF1 and ATF2, we investigated the ability of these proteins to form complexes. LEF1 formed a complex with CREB5 (Figure 5G), and TCF1 did so with endogenous ATF2 in 293T cells (Figure 5H), while coupling between endogenous LEF1 and ATF2/ATF7 was observed in Ramos and K562 cells (Figure S8). Of note, TCF4 showed weaker interaction with CREB5 compared to TCF1 (Figure S7B), suggesting that TCF4's lack of transcriptional activity could reflect a lower binding affinity for ATF2 factors. These findings indicated that TCF1/LEF1 proteins interact physically as well as functionally with ATF2 transcription factors.

**Figure 6:**
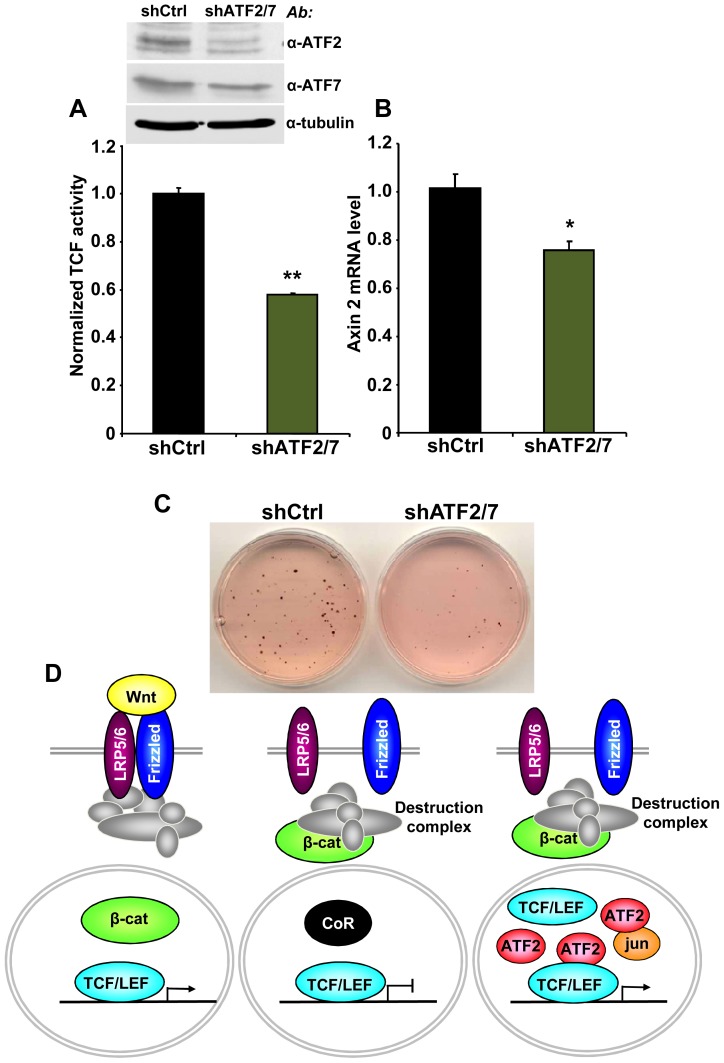
ATF2 and ATF7 knockdown in Ramos cells decreases TCF/LEF activity, Axin 2 expression and cell growth. (A–C) Ramos cells containing the TCF/LEF firefly and renilla luciferase lentiviral reporters were transduced with shATF2 and shATF7 or control lentiviral vectors. ATF2 and ATF7 down-regulation assessed by immunoblot analysis. (A) shATF2/7 inhibits constitutive TCF/LEF reporter activity in Ramos cells. (B) Knockdown of ATF2 and ATF7 reduces the expression of the Wnt target gene Axin 2 in Ramos cells as measured by quantitative real-time PCR. The results normalized to the control represent the mean values ± SEM of three independent experiments. (*) P<0.05; (**) P<0.001 compared with control (two-way Anova test) (C) shATF2/7 inhibits Ramos cell colony formation. (D) Model for β-catenin-dependent and β-catenin-independent canonical Wnt signaling. See the text for details.

Finally, we asked whether the association between TCF1/LEF1 and ATF2 factors plays a role in the β-catenin-independent up-regulation of TCF/LEF activity identified in some human hematopoietic tumor lines. We observed that shRNA-mediated down-regulation of ATF2 and ATF7 in Ramos cells significantly inhibited their endogenous TCF/LEF reporter activity (Figure 6A), as well as the expression of the Wnt target gene Axin 2 (Figure 6B). Consistent with our results using DN-TCF4 (Figure 1), the repression of TCF/LEF transcriptional activity induced by shATF2/7 in these cells was accompanied by inhibition of colony formation (Figure 6C). Together, our results provide compelling evidence for a new mechanism of constitutive TCF/LEF activation in tumor cells, which is independent of β-catenin and involves cooperation with ATF2 transcription factors.

## Discussion

In the present study, we uncovered a novel mechanism of TCF/LEF dependent transcription that bypasses β-catenin and increases expression of Wnt target genes through interaction of TCF1/LEF1 and ATF2 transcription factors (Figure 6D). We utilized several different approaches, including shRNA and decoy constructs, as well as TCF1 mutants unable to bind to β-catenin, to demonstrate that TCF1/LEF1 possess transcriptional activity that is independent of β-catenin. Expression of these proteins was able to stimulate the TCF/LEF reporter in mammalian cells, and to trigger Wnt target genes *in vivo*, accompanied by induction of the characteristic axis-duplication phenotype in *Xenopus* embryos. We showed further that this mode of TCF/LEF activation in the absence of β-catenin stabilization occurs constitutively in some human hematopoietic tumors, where it plays a role in stimulating cell growth. Thus, this cooperation between TCF1/LEF1 and ATF2 represents an unexpected new strategy used by tumor cells to aberrantly activate TCF1/LEF1 signaling independently of β-catenin and the Wnt canonical pathway.

Several arguments support a role of TCF/LEF dependent Wnt signaling in the hematopoietic system [56], and it has recently been shown that moderate levels of canonical Wnt activation promote the function and/or the maintenance of various hematopoietic lineages [57]. However, the involvement of β-catenin in these cells is highly controversial [56]. While the knock-out of TCF1 and/or LEF1 demonstrated that these transcription factors are required for normal T and B cell development [30]–[32], β-catenin conditional deletion in the mature hematopoietic system using different CRE drivers only showed mild [37] or undetectable effects [33], [36]. In fact, using two different Wnt responsive gene reporters, Jeannet et al. demonstrated that thymocytes exhibited constitutive TCF/LEF activity, which was strongly inhibited upon knock-out of TCF1, but not β-catenin or γ-catenin [34]. In light of our present findings, it is tempting to speculate that this new mechanism of β-catenin-independent activation of TCF1/LEF1 identified by us here may allow a cell autonomous up-regulated level, required for the maintenance of certain hematopoietic lineages.

We identified three TCF1 domains involved in β-catenin-independent activity, all of which localized N-terminally of the high mobility group DNA binding domain. Whereas the region between aa 100 and 211 displayed some intrinsic transactivation properties when fused to the Gal4 DNA binding domain, the N-terminal 100 aa proved to be particularly important, albeit not sufficient, to confer transcriptional activity to TCF1. The N-terminal domain of TCF/LEF proteins has been previously associated with binding to β-catenin [14], [15], [47], and our demonstration that this region also participates in β-catenin-independent signaling may help in the interpretation of several previous observations. For example, it has been assumed that binding to β-catenin is required for the role of TCF1 in T cell development, since the defects in thymocyte maturation and survival observed in TCF1 null mice could be rescued by expression of the long TCF1 isoform, but not using a short TCF1 lacking the N-Terminal 116 aa [58]. The fact that the N-terminal 100 aa of TCF1 also participate in its β-catenin-independent transcriptional activity may help to reconcile these findings with the normal phenotype of β-catenin null thymocytes [33]–[35]. Human sebaceous tumors have been reported to contain LEF-1 N-terminal domain mutations with decreased ability to interact with β-catenin [59], and expression of a LEF1 construct lacking the first 32 aa driven by the keratin 14 promoter provoked the formation of sebaceous skin tumors in mice [60]. Our results indicate that N-terminally deleted LEF1 should retain β-catenin independent transcriptional activity, suggesting that the induction of such tumors might be due to this activity rather than to inhibition of Wnt canonical signaling.

We established that both TCF1 and LEF1 interact with ATF2 transcription factors to promote TCF/LEF activity, and inhibition of such activity using either DN-TCF4 or shATF2/7 decreased the expression of Wnt target genes, as well as lymphoma cell growth. The role of ATF2 proteins in cancer depends on cell context, as these factors have been associated with both oncogenic or tumor suppressive functions [61]. Contrary to what was previously reported for the β-catenin-TCF4 complex [28], we found that c-Jun and JunB inhibited TCF1-induced transcription, suggesting that Jun proteins may compete with TCF1/LEF for binding to ATF2. While c-Jun is an oncogene overexpressed or amplified in different types of cancer, including sarcomas [61], it has been demonstrated that conditional JunB inactivation [62], [63] or PU.1 related downregulation of both JunB and c-Jun [64] provoke myeloproliferative disorders and different types of leukemia in mice. It is tempting to speculate that decreased levels of JunB and/or c-Jun may perturb the balance between different AP1 factors, thus facilitating the interaction between ATF2 and TCF1/LEF1.

With the exception of few general target genes, including LEF1 and Axin 2, the transcriptional outcome of activated Wnt signaling depends on the cell/tissue context. Gene array experiments have identified hundreds of genes whose expression is modified by Wnt, with often little overlap between different cell models [15]. A number of variables likely contribute in determining which genes are regulated in a particular cell or tissue type, including the strength of the signal, cooperation with other pathways or transcription factors, expression of different LEF/TCF isoforms [65] and, possibly, even post-translational modifications of TCF/LEF factors [66], [67]. We showed that down-regulation of β-catenin-independent TCF/LEF activity inhibited the expression of *LEF1* and *Axin 2* genes. However, further studies will be needed to obtain a broader view of the genes regulated through this new mechanism of TCF/LEF activation and to assess similarity and differences with classical Wnt/β-catenin signaling. In a recent study, TCF1 was identified as one of the most up-regulated genes in self-renewing versus partially differentiated hematopoietic multipotential precursor cells [68]. These same authors found by ChIP-seq that TCF1 primarily binds to up-regulated genes, many of which are involved in self-renewal [68]. Yet, expression of Wnt ligands was extremely low in these cells, consistent with activation of TCF1 being independent of β-catenin and canonical Wnt signaling. The integration of this type of high-throughput dataset with those generated in other systems, in which the Wnt canonical pathway is active, may aid in dissecting the different functions of β-catenin-dependent and -independent TCF/LEF signaling.

## Materials and Methods

### Cell culture, transfection and lentivirus production

Ramos (Burkitt's lymphoma), U937 (histiocytic lymphoma), K562 (chronic myelogenous leukemia) and Jurkat (acute T-cell leukemia) cells were maintained in RPMI-1640 (Lonza) supplemented with 10% fetal bovine serum (FBS; Sigma). Human mantle cell lymphoma lines including Granta-519, HBL-2, JEKO-1 and JVM-2 were generously provided by Dr. Samir Parekh, Icahn School of Medicine at Mount Sinai, and were also cultured in this medium. AB5 (immortalized human breast epithelial), PA1 (ovarian teratocarcinoma), 293T (human embryonic kidney), NIH-3T3 (mouse fibroblasts) and Mel888 (melanoma) cells were maintained in DMEM (Lonza) supplemented with 10% FBS.

Transient transfection was performed using Fugene 6 (Roche) according to the manufacturer's instructions or with polyethylenimine (Polysciences). For lentivirus production, 293T cells were co-transfected with the lentiviral vector, pCMV Δ8.91 and pMD VSV-G plasmids. The conditioned medium containing the viral particles was collected two, three and four days after transfection, supplemented with 8 µg/ml polybrene and added to a pellet of hematopoietic tumor cells, followed by centrifugation for 1 h at 500 g, 4°C and overnight incubation at 37°C, 5% CO_2_. Two days after transduction, the cells were selected in 2 µg/ml puromycin or 10 µg/ml blasticidin.

### Primary human hematopoietic tumor cells

Primary human lymphoma samples were obtained either as part of standard excisional biopsy or from peripheral blood samples from patients at the Icahn School of Medicine at Mount Sinai with informed consent reviewed and approved by the Institutional Review Board and in accordance with the Declaration of Helsinki. Specimens were processed to viable, sterile single-cell suspensions. Briefly, lymph node tissue was diced and forced through a metal sieve in a laminar flow hood into RPMI tissue culture medium. Peripheral blood mononuclear cells or disaggregated follicular lymphoma biopsy cells were pelleted by low-speed centrifugation, resuspended in media composed of 90% fetal calf serum and 10% DMSO (Sigma), frozen slowly in the vapor phase of liquid nitrogen in multiple cryotubes, and stored in liquid nitrogen. The frozen cells were thawed and maintained for 1–3 days in RPMI-1640 supplemented with 10% fetal bovine serum prior to RNA extraction for qPCR analysis or preparation of cell lysates for analysis of uncomplexed β-catenin (see below).

### Constructs

Lentiviral TCF/LEF luciferase and GFP reporters, β-catenin and Dvl shRNAs, inducible and constitutive DN-TCF4 vectors and the SuperTop reporter (pTA-Luc vector) were previously described [8], [69]. The pOT (pGL3) reporter was kindly provided by B. Vogelstein. The Top-Glow reporter was purchased from Millipore. Human TCF4 was cloned by PCR from 293T cells into pcDNA3HA vector. Human TCF1 was cloned from CCRF-CEM cells into pcDNA3HA and pcDNA3flag vectors. TCF1 mutant (D21A; E29K) was generated using the QuikChange Site-Directed Mutagenesis Kit (Stratagene). TCF1 N-terminal deletion and TCF1-TCF4 fusion constructs were generated by PCR in pcDNA3HA. The sequence corresponding to TCF1 β-catenin binding domain (N-terminal 60 aa), aa 37–122 and aa 100–211 were cloned downstream of Gal4 DNA binding domain into the pBIND vector from the CheckMate Mammalian Two-Hybrid System (Promega). Full-length LEF1 was cloned in pCAN-myc2 and pcDNA3HA (Figure 2A) vectors. Myc-tagged β-catenin and HA-tagged Wnt3a were cloned into pCCBS vector. Human ATF2 and CREB5 were cloned into pEF-flag vector. c-Jun and Jun-B were cloned into pcDNA3HA. The ATF7 construct was kindly provided by P.J. Hamard. The lentiviral shRNA constructs targeting β-catenin, γ-catenin, ATF2 and ATF7 were generated in VIRDH-EP or VIRHD-bla vectors, using the following targeting sequences: GTACGAGCTGCTATGTTCC (β-catenin), CACCATTCCCCTGTTTGTG (γ-catenin), AGCCCTCAGGAAGTTGATTAAA (ATF2) and CGAAGAACTCACTTCTCAGAA (ATF7). All constructs were sequence verified.

### Antibodies, immunoblotting and immunoprecipitation

The following antibodies were purchased from commercial sources: mouse anti-β-catenin, mouse anti-γ-catenin (BD Biosciences), mouse anti-tubulin, mouse anti-Flag, mouse anti-HA (Sigma), rabbit anti-ATF2, rabbit anti-HA (Santa Cruz), goat anti-Axin (R&D Systems), rabbit anti-ATF7 (Abcam), mouse anti-LEF1 (Millipore). Mouse anti-myc clone 9E10 was obtained from the Mount Sinai Hybridoma Core Facility.

For immunoblot, cells were washed once with phosphate-buffered saline (PBS) and lysed on ice in lysis buffer containing 50 mM Hepes pH 7.6, 150 mM NaCl, 5 mM EDTA, 1% Nonidet P-40, 20 mM NaF, 2 mM sodium orthovanadate, supplemented with the Complete Mini proteinase inhibitor cocktail tablets (Roche). Lysates were cleared by centrifugation at 20,000× g for 15 min at 4°C and protein concentrations were determined by using the Bio-Rad protein assay (Bio-Rad). Sodium dodecyl sulfate (SDS) loading buffer was added to equal amounts of lysate, followed by SDS-polyacrylamide gel electrophoresis (SDS-PAGE) and transfer to Immobilon-P membranes (Millipore). Antibodies used in immunoblot analysis were revealed by chemiluminescence using ECL Western Blotting Substrate (Thermo Fisher Scientific) or using the Odyssey Infrared Imaging System (LI-COR Biotechnology). For immunoprecipitation, equal amounts of cell lysates were incubated either with anti-Flag M2 agarose beads (Sigma) for 3 hrs at 4°C or with mouse anti-myc or mouse anti-HA antibodies for 1 hr at 4°C, followed by 3 hr incubation with Protein G Sepharose 4 Fast Flow beads (GE Healthcare). For LEF1 immunoprecipitation, equal amounts of cell lysates were incubated with aLEF1 antibody for 2 hrs at 4°C, followed by overnight incubation with Protein G Sepharose 4 Fast Flow beads. Beads were washed three times with lysis buffer and resuspended in SDS loading buffer, followed by SDS-PAGE and immunoblot.

### Free β-catenin and reporter assays

Uncomplexed β-catenin was measured using glutathione S-transferase (GST)–E-cadherin or GST-TCF1 beads as described previously [70]. Briefly, bacterially expressed E-cadherin or TCF1 β-catenin binding domains fused to GST were bound to glutathione-Sepharose beads (GE Healthcare) and incubated with equal amounts of cell lysates. After pull-down, the samples were subjected to immunoblot analysis using anti-β-catenin antibody. For luciferase reporter assay, the cells were transfected or transduced with the TCF/LEF firefly luciferase reporters and the renilla luciferase control reporters or transfected with the Gal4 responsive vector pG5luc (Promega) and the renilla containing pBind plasmid. The cells were lysed and luciferase activity was measured using the Dual-Luciferase Reporter Assay System (Promega) according to the manufacturer's instructions. For GFP reporter assay, cells transduced with the Top or Fop TCF/LEF GFP reporter were transferred to polystyrene tubes (Falcon) and subjected to FACS analysis (FACScan; Becton Dickinson) using CellQuest 3.2 software (Becton Dickinson).

### Colony forming assay

Growth of hematopoietic tumor cells in soft agarose was determined by seeding 2×10^3^ cells per 60-mm dish in 0.5% sea plaque agarose (Cambrex) in RPMI supplemented with 10% FBS on a semisolid bottom layer of growth medium containing 1% agarose. Cells were fed once weekly with 0.3 mL of medium and stained after 17 days with iodonitrotetrazolium (Sigma). The cells containing Tet-off inducible DN-TCF4-GFP vector were grown overnight in the presence or the absence of 100 ng/ml doxycycline, followed by extensive washing in PBS. The cells were then counted and seeded in soft agarose dishes in the presence or the absence of 100 ng/ml doxycyline. The remaining cells were grown for one additional day with or without 100 ng/ml doxycyline, and FACS analysis was performed to assess the level of induction of DN-TCF4-GFP.

### qPCR

Total RNA was extracted using the Trizol Reagent (Invitrogen), incubated with DNase I (Invitrogen) and reverse transcribed in the presence of random primers using SuperScript II reverse transcriptase (Invitrogen) according to the manufacturer's protocol. Quantitative PCR was performed using FastStart SYBR Green Master (Roche) on a MJ Opticon (Bio-Rad). The primers used for qPCR were: TATA Binding Protein (5′-ATCAGTGCCGTGGTTCGT and 5′-TTCGGAGAGTTCTGGGATTG), 18S (5′-GTAACCCGTTGAACCCAT and 5′-CCATCCAATCGGTAGTAG), Axin 2 (5′-ACTGCCCACACGATAAGGAG and 5′-CTGGCTATGTCTTTGGACCA), LEF1 (5′-CTTTATCCAGGCTGGTCTGC and 5′-TCGTTTTCCACCATGTTTCA).

### *Xenopus* experiments

RNA was synthesized in vitro in the presence of cap analog using the mMessage mMachine kit (Ambion). Microinjection, explant dissection, cell culture and whole-mount antibody staining were performed as described [71]. The 12/101 antibody (Developmental Studies Hybridoma Bank, University of Iowa) was used at a 1∶1 dilution. Secondary antibody was a donkey anti-mouse IgG coupled to horseradish peroxidase (Jackson Laboratories), and was used at 1∶1000 dilution. Color reactions were performed using the Vector SG kit (Vector Laboratories). For RT-PCR, *Xenopus laevis* embryos were staged and harvested at appropriate stages according to morphological criteria. RNA was prepared using RNA Bee RNA isolation reagent (Tel-Test Inc.). RT-PCR was performed as described [72]. Primers used in this study are as follows: Xbrachyury (5′-GGATCGTTATCACCTCTG and 5′-GTGTAGTCTGTAGCAGCA), chordin (5′-CAGTCAGATGGAGCAGGATC and 5′-AGTCCCATTGCCCGAGTTGC), EF1-α (5′-CAGATTGGTGCTGGATATGC and 5′-ACTGCCTTGATGACTCCTAG), Xnr3 (5′-GTGAATCCACTTGTGCAGTT and 5′-ACAGAGCCAATCTCATGTGC).

Studies on Xenopus laevis embryos were performed in accordance with the guidelines of the American Veterinary Medical Association, and under the auspices of the Queens College Institutional Animal Care and Use Committee (IACUC). All experiments were undertaken with the highest regard for scientific, ethical, and humane principles.

## Supporting Information

Figure S1 Endogenous TCF/LEF activation in hematopoietic cancer cells. (A) K562 and U937 cells were transduced with wild-type (Top) or mutant (Fop) TCF/LEF lentiviral reporter driving the expression of GFP, followed by FACS analysis. Breast MDAMB157 and lung H23 cells, which display constitutive autocrine Wnt activation, were used as positive controls. (B) β-catenin or γ-catenin knockdown in K562 cells did not affect TCF/LEF activity. K562 cells containing Top or Fop luciferase lentiviral reporter were transduced with β-catenin or γ-catenin shRNAs, and the TCF/LEF reporter activity was measured (lower panel). β-catenin and γ-catenin down-regulation was assessed by immunoblot (upper panel). (C) K562 cells were transduced with Top- or Fop-GFP lentiviral reporter, followed by transduction with luciferase (control) or DN-TCF4 lentiviruses. The GFP levels, corresponding to TCF/LEF activation, were measured by FACS. (D) The mRNA levels of Lef1 and Axin 2 in the cells depicted in C were quantified by real-time PCR, and the expression of DN-TCF4 was assessed by immunoblot. Bcl-xL was used as a loading control. (E) The indicated mantle cell lymphoma (MCL) cells were transduced with Top or Fop luciferase reporter and a renilla luciferase virus, and the TCF/LEF transcriptional activity was calculated by dividing the Top/renilla ratio by the Fop/renilla ratio. Ramos and U937 cells were used as positive and negative controls, respectively. (F) Lack of uncomplexed β-catenin in the MCL cells used in E, as measured by GST-TCF1 pull-down. Ovarian PA1 cells were used as positive control, while Ramos and U937 cells were used as negative controls. (G) Lack of uncomplexed β-catenin in primary hematopoietic tumors. The type of tumor corresponding to each sample is indicated in Figure 1G.
(PDF)

Figure S2 TCF1 expression triggers TCF/LEF reporter activity in 293T cells. (A) 293T cells were co-transfected with the indicated wild-type (T) or mutant (F) TCF/LEF reporters, the renilla plasmid pRL-CMV and increasing amounts of TCF1 construct or empty pcDNA3HA vector (500 ng). The TCF/LEF activity is expressed as relative luciferase units normalized by the renilla luciferase reading. (B) 293T cells were co-transfected with SuperTop reporter, pBind renilla plasmid and HA-tagged TCF1 in the absence or the presence of ten-fold amount of HA-tagged DN-TCF4. Two days after transfection, the cells were lysed, followed by luciferase assay (lower panel) and immunoblot using anti-HA antibody to assess the expression levels of TCF1 and DN-TCF4 (upper panel). (C) Empty vector, TCF1, LEF1 or TCF4 constructs were co-transfected with SuperTop or SuperFop and renilla luciferase plasmids in 293T cells and the Top/Fop ratio was calculated.
(PDF)

Figure S3 Lack of physical and functional interaction of D21A;E29K mutant TCF1 (TCF1mt) with β-catenin or γ-catenin. (A) 293T cells were transfected as indicated with myc-tagged β-catenin and HA-tagged wild-type or mutant TCF1, followed by immunoprecipitation using anti-myc antibody and immunoblot with anti-myc or anti-HA antibodies. (B) Mel888 melanoma cells containing a β-catenin mutation resulting in constitutive Wnt activation were co-transfected with SuperTop reporter, the renilla pRL-CMV plasmid and equal amounts of empty vector, TCF1, d36TCF1 or TCF1mt. Two days after transfection, luciferase assay was performed and the TCF/LEF reporter activity is represented as renilla normalized relative luciferase units. (C) 293T cells were co-transfected with SuperTop reporter, the renilla pRL-CMV plasmid, γ-catenin and equal amounts of empty vector, TCF1 or TCF1mt, followed by luciferase assay two days after transfection.
(PDF)

Figure S4 Exon IVa is not required for TCF1 transcriptional activity. 293T cells were co-transfected with the SuperTop or SuperFop reporter, the renilla plasmid pRL-CMV and the indicated TCF1 or TCF4 constructs. Two days after transfection, luciferase assay was performed and TCF/LEF activity expressed as the Top/Fop ratio of the renilla normalized luciferase values.
(PDF)

Figure S5 aa 101–211 are involved in β-catenin-independent, but not β-dependent, TCF1 transcriptional activity. (A) 293T cells were co-transfected with the indicated SuperTop reporter, the renilla plasmid pRL-CMV and the indicated TCF1 construct followed two days later by luciferase assay. The TCF/LEF activity is expressed as relative luciferase units normalized by the renilla luciferase reading (lower panel). The expression of the different TCF1 constructs was assessed by immunoblot using the same cell lysates (upper panel). (B) 293T cells were co-transfected with SuperTop reporter, pRL-CMV and β-catenin (S33Y) with or without the indicated TCF1 constructs, followed two days later by luciferase assay.
(PDF)

Figure S6 ATF2 synergizes with TCF1mt independently of β-catenin. 293T cells were co-transfected with TCF1mt, ATF2 or Wnt3a in combination with shRNA vectors targeting either CHK1 (control), the three Dvl isoforms or β-catenin, in the presence of the SuperTop reporter and the renilla plasmid pRL-CMV. Three days after transfection, luciferase assay was performed and TCF/LEF activity expressed as the mean +/− SD of the renilla normalized luciferase values. The Wnt3a construct was used to assess the efficiency of shDvl and shβ-catenin in inhibiting Wnt canonical signaling.
(PDF)

Figure S7 Lack of synergy between TCF4 and ATF2 factors. (A) 293T cells were co-transfected with TCF4, ATF2, CREB5 or ATF7 as indicated, in the presence of the SuperTop reporter and the renilla plasmid pRL-CMV. Two days after transfection, luciferase assay was performed and TCF/LEF activity expressed as the mean +/− SD of the renilla normalized luciferase values (lower panel). The same lysate was used to assess the expression of TCF4 and ATF2 factors by immunoblot (upper panel). (B) 293T cells were co-transfected as indicated with Flag-tagged CREB5, HA-tagged TCF1 and HA-tagged TCF4, followed by immunoprecipitation using anti-Flag antibody and immunoblot with anti-HA or anti-Flag antibodies.
(PDF)

Figure S8 Interaction between endogenous LEF1 and ATF2/7 in hematopoietic tumor cells. 2 mg of Lysate from K562 (A) or Ramos (B) cells was immunoprecipitated using 10 ug of anti-LEF1 or control antibody, followed by immunoblot using the indicated antibodies. The band corresponding to immunoprecipitated LEF1 (∼55 kDa) was masked by the IgG heavy chain used for the immunoprecipitation.
(PDF)
