# Supplementary figures and images for: β-Catenin-Independent Activation of TCF1/LEF1 in Human Hematopoietic Tumor Cells through Interaction with ATF2 Transcription Factors
Source: PLoS Genet. 2013 Aug 15;9(8):e1003603. doi: 10.1371/journal.pgen.1003603 (PMC3744423; doi:10.1371/journal.pgen.1003603)

**A**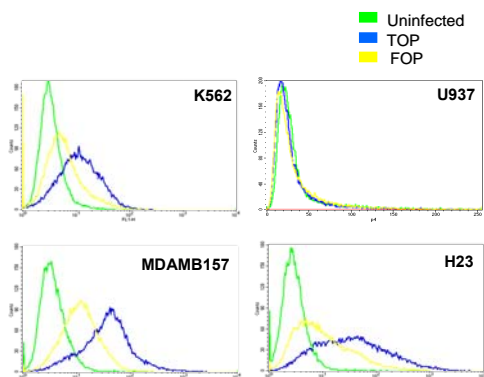**B**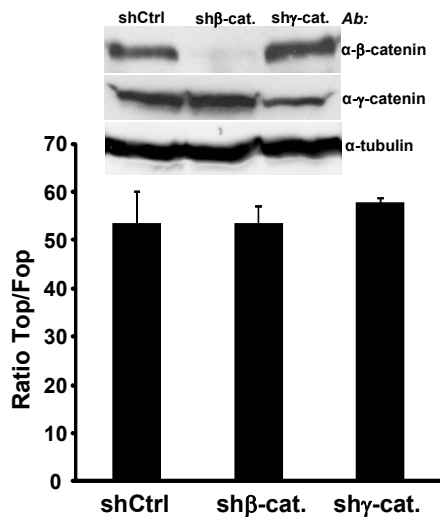**C**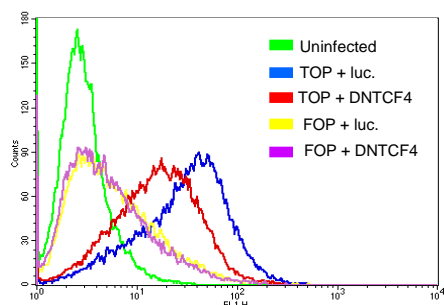**D**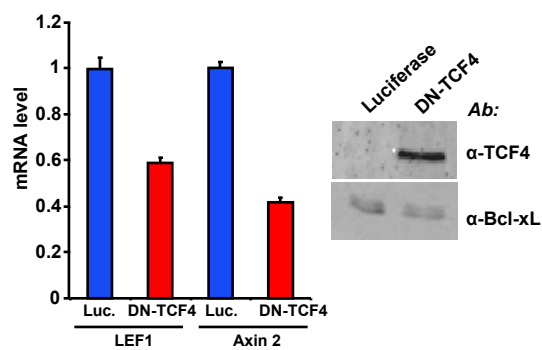**E**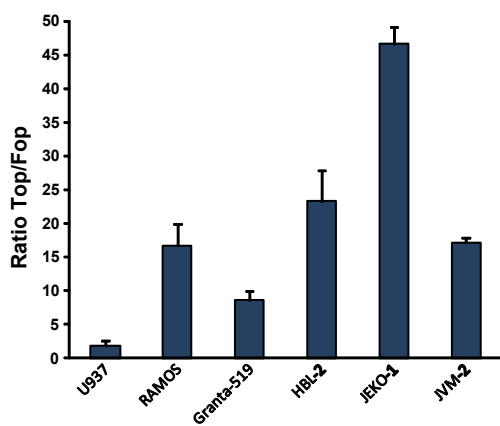**F**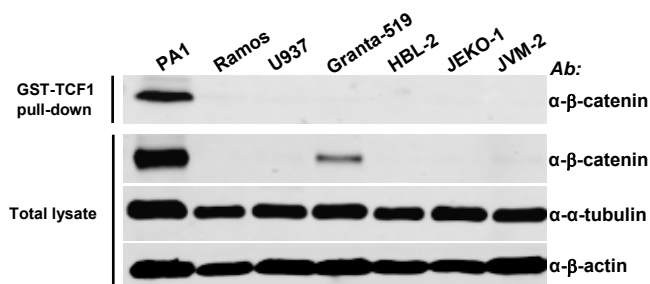**G**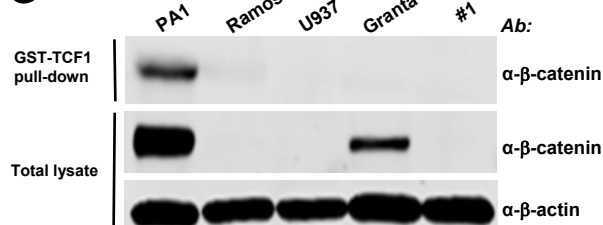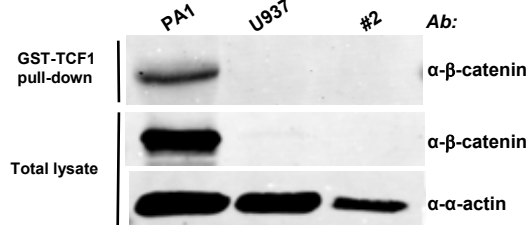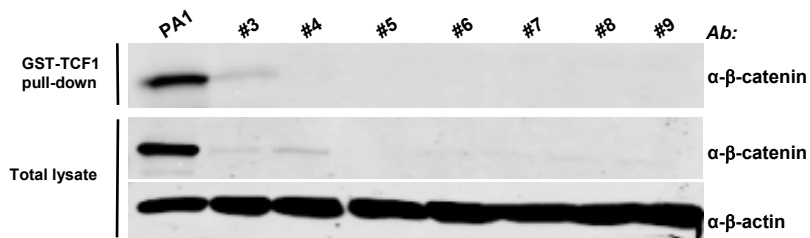

Supplement: Figure S1 — Endogenous TCF/LEF activation in hematopoietic cancer cells. (A) K562 and U937 cells were transduced with wild-type (Top) or mutant (Fop) TCF/LEF lentiviral reporter driving the expression of GFP, followed by FACS analysis. Breast MDAMB157 and lung H23 cells, which display constitutive autocrine Wnt activation, were used as positive controls. (B) β-catenin or γ-catenin knockdown in K562 cells did not affect TCF/LEF activity. K562 cells containing Top or Fop luciferase lentiviral reporter were transduced with β-catenin or γ-catenin shRNAs, and the TCF/LEF reporter activity was measured (lower panel). β-catenin and γ-catenin down-regulation was assessed by immunoblot (upper panel). (C) K562 cells were transduced with Top- or Fop-GFP lentiviral reporter, followed by transduction with luciferase (control) or DN-TCF4 lentiviruses. The GFP levels, corresponding to TCF/LEF activation, were measured by FACS. (D) The mRNA levels of Lef1 and Axin 2 in the cells depicted in C were quantified by real-time PCR, and the expression of DN-TCF4 was assessed by immunoblot. Bcl-xL was used as a loading control. (E) The indicated mantle cell lymphoma (MCL) cells were transduced with Top or Fop luciferase reporter and a renilla luciferase virus, and the TCF/LEF transcriptional activity was calculated by dividing the Top/renilla ratio by the Fop/renilla ratio. Ramos and U937 cells were used as positive and negative controls, respectively. (F) Lack of uncomplexed β-catenin in the MCL cells used in E, as measured by GST-TCF1 pull-down. Ovarian PA1 cells were used as positive control, while Ramos and U937 cells were used as negative controls. (G) Lack of uncomplexed β-catenin in primary hematopoietic tumors. The type of tumor corresponding to each sample is indicated in Figure 1G. (PDF) [file pgen.1003603.s001.pdf]

**A**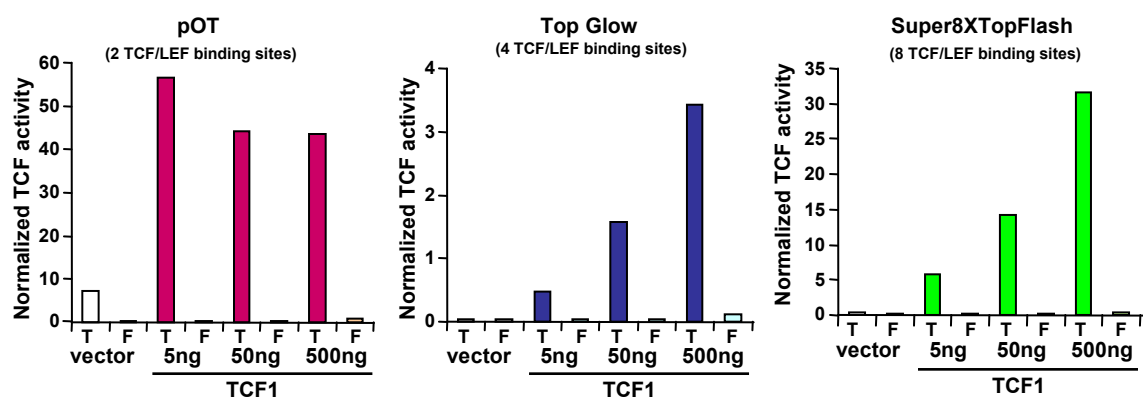**B**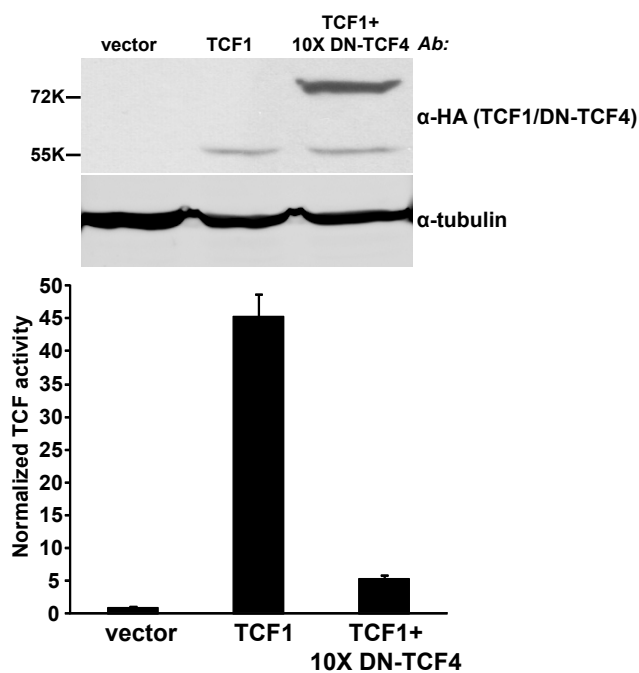**C**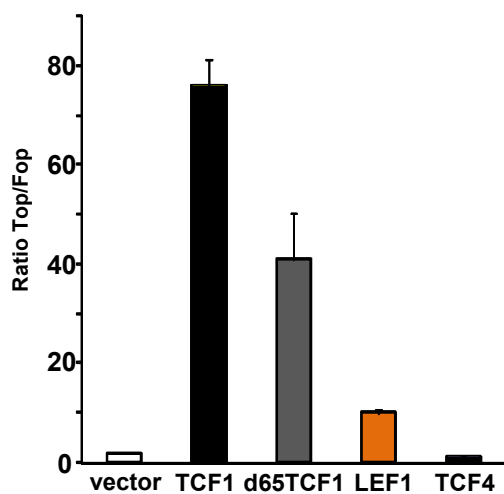

Supplement: Figure S2 — TCF1 expression triggers TCF/LEF reporter activity in 293T cells. (A) 293T cells were co-transfected with the indicated wild-type (T) or mutant (F) TCF/LEF reporters, the renilla plasmid pRL-CMV and increasing amounts of TCF1 construct or empty pcDNA3HA vector (500 ng). The TCF/LEF activity is expressed as relative luciferase units normalized by the renilla luciferase reading. (B) 293T cells were co-transfected with SuperTop reporter, pBind renilla plasmid and HA-tagged TCF1 in the absence or the presence of ten-fold amount of HA-tagged DN-TCF4. Two days after transfection, the cells were lysed, followed by luciferase assay (lower panel) and immunoblot using anti-HA antibody to assess the expression levels of TCF1 and DN-TCF4 (upper panel). (C) Empty vector, TCF1, LEF1 or TCF4 constructs were co-transfected with SuperTop or SuperFop and renilla luciferase plasmids in 293T cells and the Top/Fop ratio was calculated. (PDF) [file pgen.1003603.s002.pdf]

**A**

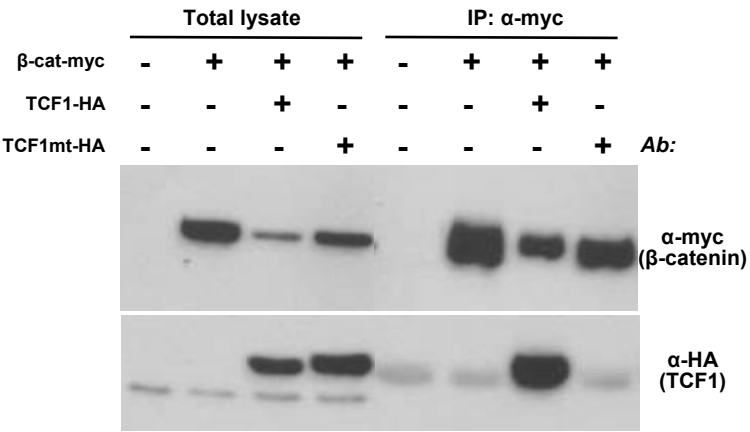

**B**

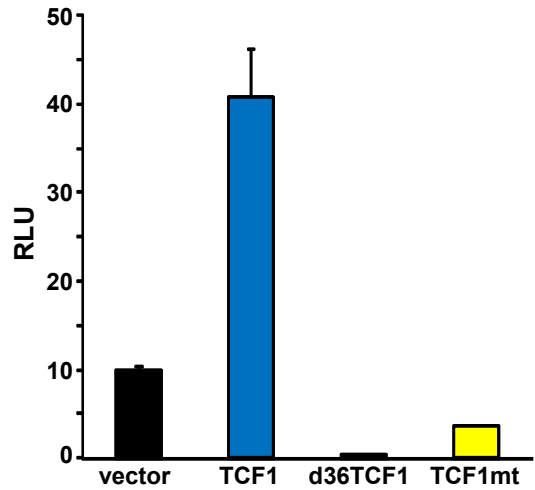

**C**

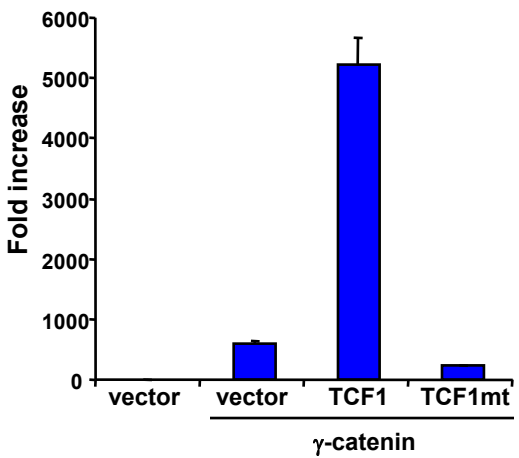

Supplement: Figure S3 — Lack of physical and functional interaction of D21A;E29K mutant TCF1 (TCF1mt) with β-catenin or γ-catenin. (A) 293T cells were transfected as indicated with myc-tagged β-catenin and HA-tagged wild-type or mutant TCF1, followed by immunoprecipitation using anti-myc antibody and immunoblot with anti-myc or anti-HA antibodies. (B) Mel888 melanoma cells containing a β-catenin mutation resulting in constitutive Wnt activation were co-transfected with SuperTop reporter, the renilla pRL-CMV plasmid and equal amounts of empty vector, TCF1, d36TCF1 or TCF1mt. Two days after transfection, luciferase assay was performed and the TCF/LEF reporter activity is represented as renilla normalized relative luciferase units. (C) 293T cells were co-transfected with SuperTop reporter, the renilla pRL-CMV plasmid, γ-catenin and equal amounts of empty vector, TCF1 or TCF1mt, followed by luciferase assay two days after transfection. (PDF) [file pgen.1003603.s003.pdf]

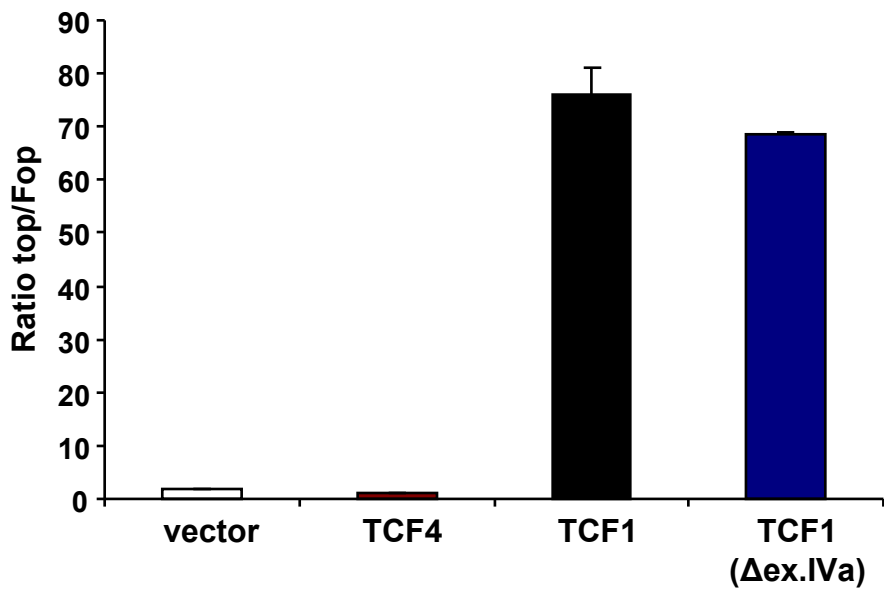

Supplement: Figure S4 — Exon IVa is not required for TCF1 transcriptional activity. 293T cells were co-transfected with the SuperTop or SuperFop reporter, the renilla plasmid pRL-CMV and the indicated TCF1 or TCF4 constructs. Two days after transfection, luciferase assay was performed and TCF/LEF activity expressed as the Top/Fop ratio of the renilla normalized luciferase values. (PDF) [file pgen.1003603.s004.pdf]

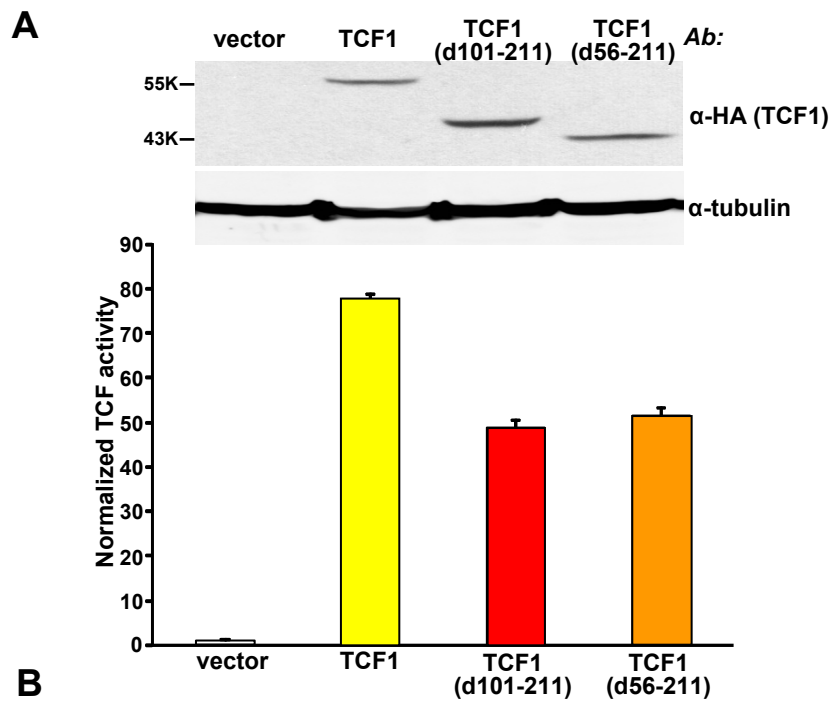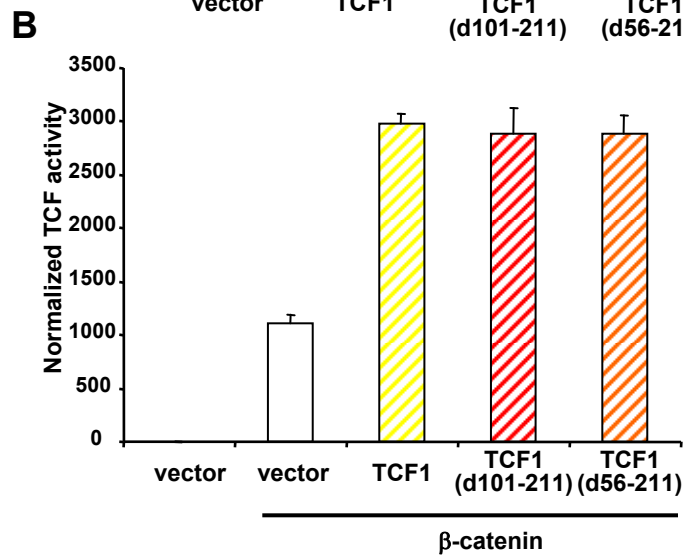

Supplement: Figure S5 — aa 101–211 are involved in β-catenin-independent, but not β-dependent, TCF1 transcriptional activity. (A) 293T cells were co-transfected with the indicated SuperTop reporter, the renilla plasmid pRL-CMV and the indicated TCF1 construct followed two days later by luciferase assay. The TCF/LEF activity is expressed as relative luciferase units normalized by the renilla luciferase reading (lower panel). The expression of the different TCF1 constructs was assessed by immunoblot using the same cell lysates (upper panel). (B) 293T cells were co-transfected with SuperTop reporter, pRL-CMV and β-catenin (S33Y) with or without the indicated TCF1 constructs, followed two days later by luciferase assay. (PDF) [file pgen.1003603.s005.pdf]

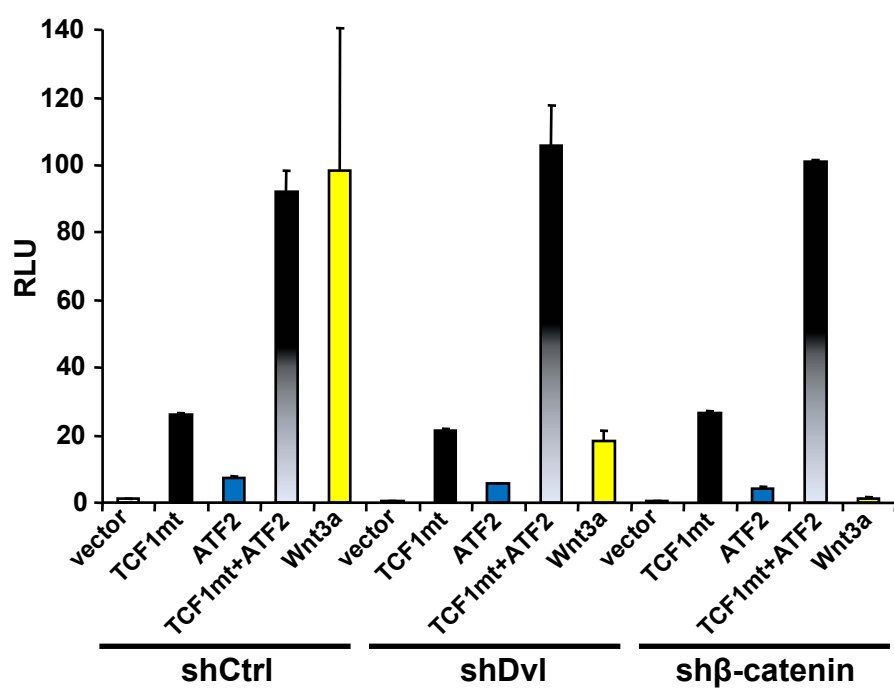

Supplement: Figure S6 — ATF2 synergizes with TCF1mt independently of β-catenin. 293T cells were co-transfected with TCF1mt, ATF2 or Wnt3a in combination with shRNA vectors targeting either CHK1 (control), the three Dvl isoforms or β-catenin, in the presence of the SuperTop reporter and the renilla plasmid pRL-CMV. Three days after transfection, luciferase assay was performed and TCF/LEF activity expressed as the mean +/− SD of the renilla normalized luciferase values. The Wnt3a construct was used to assess the efficiency of shDvl and shβ-catenin in inhibiting Wnt canonical signaling. (PDF) [file pgen.1003603.s006.pdf]

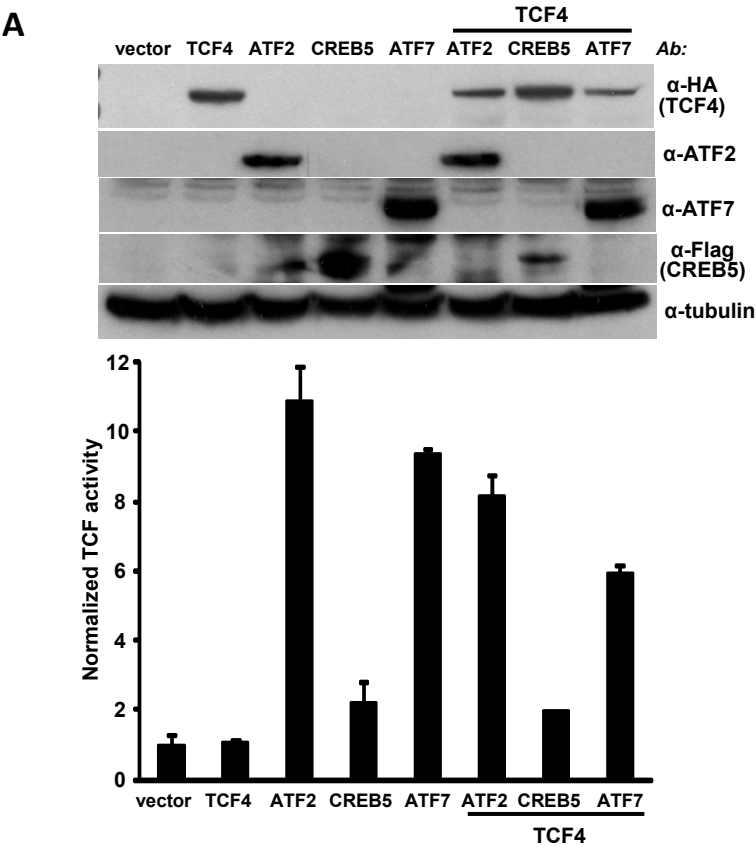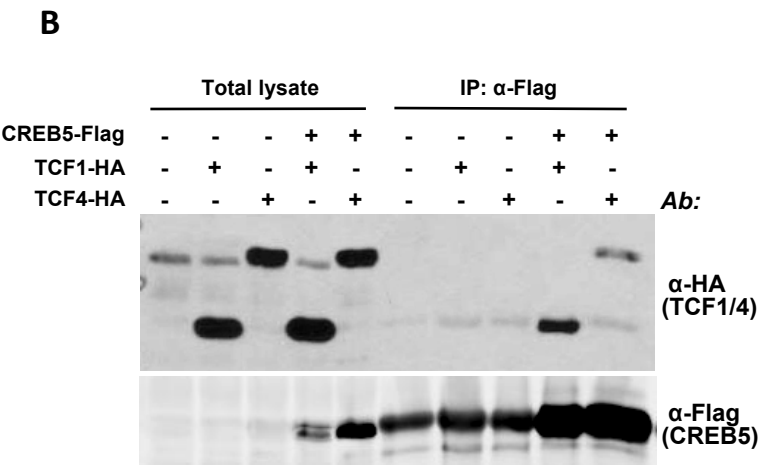

Supplement: Figure S7 — Lack of synergy between TCF4 and ATF2 factors. (A) 293T cells were co-transfected with TCF4, ATF2, CREB5 or ATF7 as indicated, in the presence of the SuperTop reporter and the renilla plasmid pRL-CMV. Two days after transfection, luciferase assay was performed and TCF/LEF activity expressed as the mean +/− SD of the renilla normalized luciferase values (lower panel). The same lysate was used to assess the expression of TCF4 and ATF2 factors by immunoblot (upper panel). (B) 293T cells were co-transfected as indicated with Flag-tagged CREB5, HA-tagged TCF1 and HA-tagged TCF4, followed by immunoprecipitation using anti-Flag antibody and immunoblot with anti-HA or anti-Flag antibodies. (PDF) [file pgen.1003603.s007.pdf]

**A**

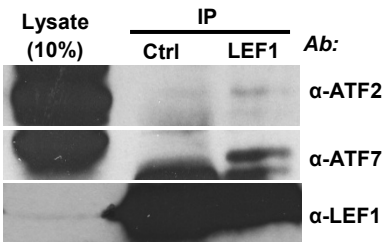

*K562 cells*

**B**

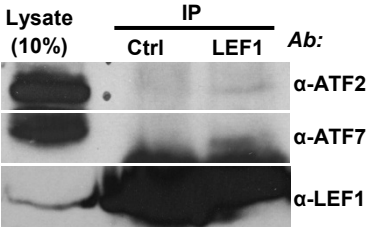

*Ramos cells*

Supplement: Figure S8 — Interaction between endogenous LEF1 and ATF2/7 in hematopoietic tumor cells. 2 mg of Lysate from K562 (A) or Ramos (B) cells was immunoprecipitated using 10 ug of anti-LEF1 or control antibody, followed by immunoblot using the indicated antibodies. The band corresponding to immunoprecipitated LEF1 (∼55 kDa) was masked by the IgG heavy chain used for the immunoprecipitation. (PDF) [file pgen.1003603.s008.pdf]
